# Surface Proton Conduction of Sm-Doped CeO_2-*δ*_ Thin Film Preferentially Grown on Al_2_O_3_ (0001)

**DOI:** 10.1186/s11671-020-3267-5

**Published:** 2020-02-17

**Authors:** D. Nishioka, T. Tsuchiya, W. Namiki, M. Takayanagi, K. Kawamura, T. Fujita, R. Yukawa, K. Horiba, H. Kumigashira, T. Higuchi

**Affiliations:** 1 Department of Applied Physics, Tokyo University of Science, Katsushika, Tokyo, 125-8585 Japan; 2 International Center for Materials Nanoarchitectonics (WPI-MANA), National Institute for Materials Science (NIMS), Tsukuba, Ibaraki, 305-0044 Japan; 3 Photon Factory, High Energy Accelerator Organization (KEK), Tsukuba, Ibaraki, 305-0801 Japan; 4 Institute of Multidisciplinary Research for Advanced Materials (IMRAM), Tohoku University, Sendai, 980-8577 Japan

**Keywords:** Sm-doped CeO_2_ (SDC), Thin film, Mixed valence state, Surface proton conduction, O–H bond

## Abstract

Sm-doped CeO_2-*δ*_ (Ce_0.9_Sm_0.1_O_2-*δ*_; SDC) thin films were prepared on Al_2_O_3_ (0001) substrates by radio frequency magnetron sputtering. The prepared thin films were preferentially grown along the [111] direction, with the spacing of the (111) plane (*d*_111_) expanded by 2.6% to compensate for a lattice mismatch against the substrate. The wet-annealed SDC thin film, with the reduced *d*_111_ value, exhibited surface protonic conduction in the low-temperature region below 100 °C. The O1*s* photoemission spectrum exhibits H_2_O and OH^−^ peaks on the SDC surface. These results indicate the presence of physisorbed water layers and the generation of protons on the SDC (111) surface with oxygen vacancies. The protons generated on the SDC surface were conducted through a physisorbed water layer by the Grotthuss mechanism.

## Background

Fluorite-type CeO_2-*δ*_ oxides are good solid electrolyte candidates for solid oxide fuel cells (SOFC) due to their high oxygen ion conductivity in high-temperature (HT) regions above 800 °C [1–7]. In particular, the oxygen ion conduction of CeO_2-*δ*_ bulk crystal can be tuned by substituting trivalent rare-earth cations [7–9], while small electron conduction also prevails under low-oxygen potential conditions owing to the formation of hopping electrons on cation sites through the propagation of oxygen nonstoichiometry [10–13].

Recently, high proton conductivity was observed for porous and nanocrystalline CeO_2-*δ*_ and Y-stabilized ZrO_2_ (YSZ) below 100 °C, including the room temperature region [14–25]. Although the detailed mechanism is still an open question, such conduction is believed to result from the surface adsorption of water molecules. Protons are generated by adsorbed water molecules and conducted through adsorbed water layers. This means that a large surface area is crucial to increase the proton conduction. When considering practical devices, thin film forms are more suitable than porous or nanocrystalline forms. Proton conducting CeO_2_ thin films may be applied to many types of electrochemical devices, such as electric double-layer transistors (EDLTs), which operate based on EDL-induced carrier doping [26–30]. While surface proton conduction has already been found in both pure and doped CeO_2_ ceramics and thin films [18–22], the proton conductivity was not sufficiently high for practical applications.

In this study, in order to improve CeO_2_ thin film surface proton conductivity, we prepared a preferentially oriented Sm-doped CeO_2_ (SDC) thin film on an Al_2_O_3_ (0001) substrate and investigated its surface proton conductivity.

## Methods/Experimental

### Preparation of SDC Thin Film

A 10-mol% Sm-doped CeO_2_ ceramic target was synthesized by a solid-state reaction method. CeO_2_ (99.9%, Furuuchi Chem. Coop.) and Sm_2_O_3_ (99.99%, Furuuchi Chem. Coop.) powders were ball-milled for 24 h, after which the mixture was pressed into a disk shape at 50 MPa and sintered in air at 1250 °C for 6 h. The SDC thin films were deposited on Al_2_O_3_ (0001) substrates by radio frequency (RF) magnetron sputtering using a ceramic target. The RF magnetron sputtering system was arranged in a symmetric configuration, with a rotating substrate holder for compositional uniformity, and was kept at a base pressure of 2.0 × 10^−7^ Torr. The distance between the target and the substrates was 70 mm. The ceramic target RF power and the Ar gas flow rate were set at 50 W and 30 sccm, respectively. The deposition pressure and the substrate temperature were fixed at 8.0 × 10^−3^ Torr and 700 °C, respectively. The SDC thin film was annealed in a wet atmosphere (Ar:O_2_ = 4:1, *p*(H_2_O) = 2.3 kPa) at 500 °C for 1 h. From the Ce 3*d*, Sm3*d*, and O1*s* core level photoemission spectroscopy (PES) spectra, the composition of the SDC thin film was calculated to be Ce_0.858_Sm_0.142_O_1.912_.

### Characterizations of the Crystalline and Conductivity

The crystalline quality of the thin films was characterized by X-ray diffraction (XRD) with CuKα using a Rigaku Miniflex 600. The electrical conductivities were characterized by the AC impedance method, using a frequency response analyzer (Solartron 1260) and an amplifier (Solartron 1296), in a temperature region in dry air (Ar:O_2_ = 4:1) and wet air (Ar:O_2_ = 4:1, *p*(H_2_O) = 2.3 kPa). To measure the in-plane electrical conductivity, a ~ 100-nm-thick interdigital Ag electrode was deposited on the film surface through a metal shadow mask by sputtering. The area of the thin film was 8.0 × 8.0 mm^2^. The length and width of the conducting path were 45.0 mm and 0.4 mm, respectively [15]. The conducting carrier was estimated from the electrical conductivity against the *P*_O2_ (not shown). The measurement of the electrical conductivity frequency region was changed from 32 to 100 MHz. The conductivity value at each temperature was carefully calculated by taking the resistance, the conductivity path, and a cross-section area of the thin film.

### Characterization of the Electronic Structure

The electronic structures were measured by photoemission spectroscopy (PES) and X-ray absorption spectroscopy (XAS). The spectroscopic measurements were conducted at the KEK Photon Factory BL-2A MUSASHI undulator beamline [31]. The XAS spectrum was recorded in a total electron yield mode. PES spectra were acquired using a VG-Scienta SES-2002 hemispherical analyzer. The PES and XAS resolutions were set at approximately 100 and 80 meV, respectively.

## Results and Discussion

**Fig. 1:**
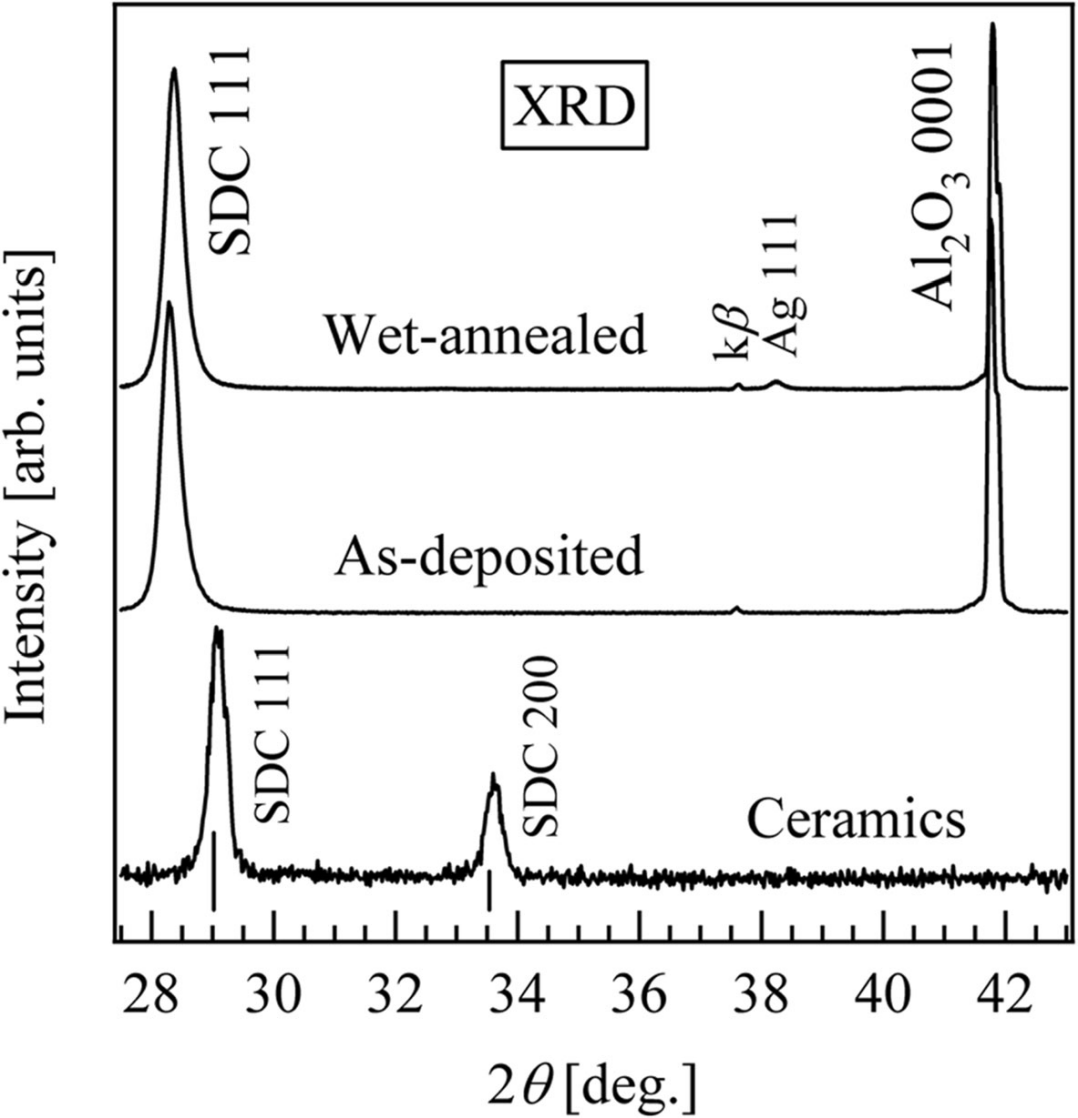
XRD patterns of the as-deposited, wet-annealed SDC thin films and SDC ceramic. The two solid vertical lines are the CeO_2_ (111) and (200) planes

Figure 1 shows the XRD patterns of the SDC ceramic, as-deposited and wet-annealed SDC thin films. The SDC ceramic target was polycrystalline, and the thin film was preferentially grown along the [111] direction. For this study, we prepared a nanocrystalline SDC ceramic which, while exhibiting admittedly poor crystallinity, did exhibit sufficient proton conductivity to allow us to discuss the differences between the SDC ceramic and thin film. The positions of the 111 peak of the SDC ceramic and as-deposited thin film are at ~ 29.02° and ~ 28.31°, and the calculated spacing of the (111) plane (*d*_111_) is 3.070 and 3.151 Å, respectively. The *d*_111_ of the thin film was expanded by 2.6% from that of the ceramic target, so as to compensate for the lattice mismatch between SDC and Al_2_O_3_. In addition, at 3.091 Å, the *d*_111_ of the wet-annealed thin film was 1.9% less than that of the as-deposited thin film. This shrinkage of *d*_111_ is due to the chemical absorption of water molecules by oxygen vacancies through wet annealing, as in the following reaction [32]:

1 $$ {\mathrm{H}}_2\mathrm{O}+{\mathrm{V}}_{\mathrm{O}}^{\bullet \bullet }+\frac{1}{2}{\mathrm{O}}_2\to 2{\left(\mathrm{OH}\right)}^{\bullet } $$

A weak wet annealing peak, at ~ 38.0°, is assigned to the 111 peak of the Ag electrode used for the conductivity measurement.

**Fig. 2:**
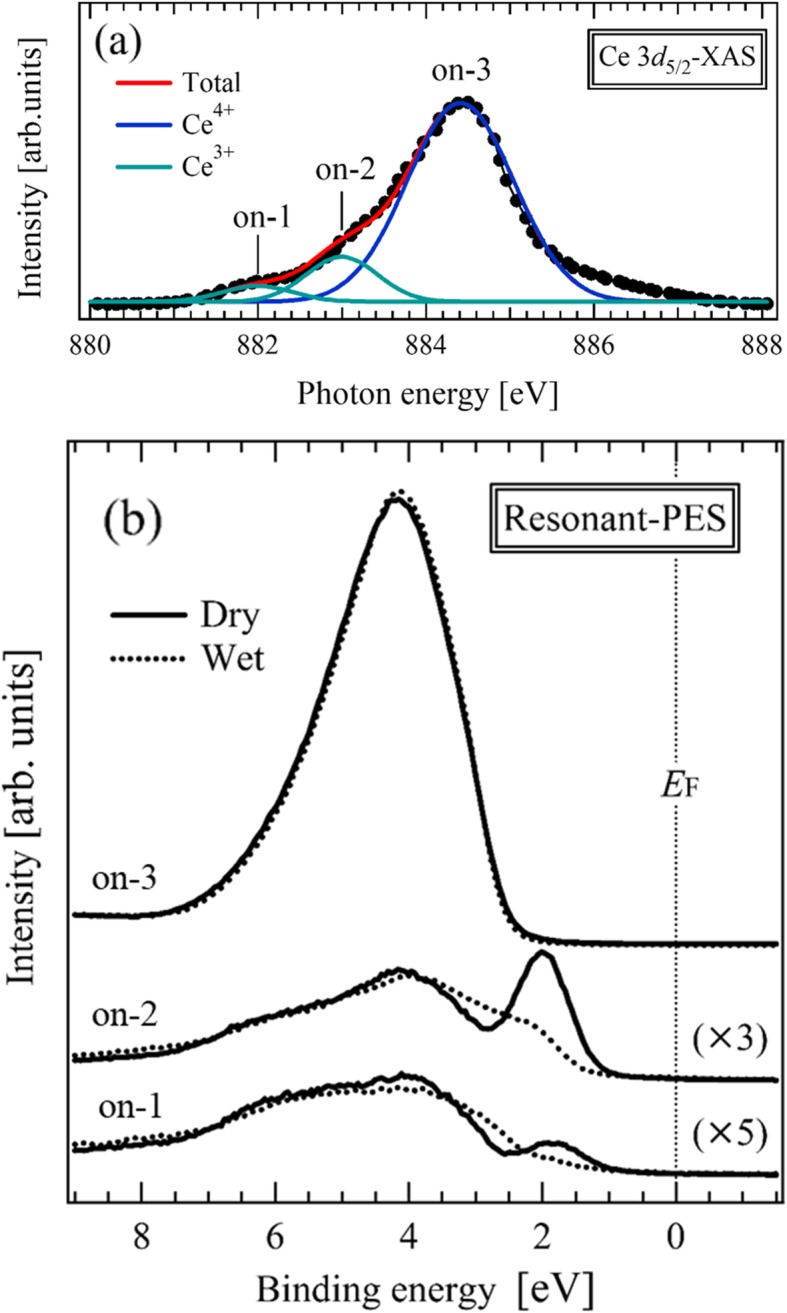
**a** Ce 3*d* XAS spectrum of the as-deposited SDC thin film. The labels on-1, on-2, and on-3 indicate the excitation energies for the resonant PES measurements. **b** Resonant PES spectra of the as-deposited and wet-annealed SDC thin films measured at on-1, on-2, and on-3 in **a**. The green and blue curves are the Ce^3+^ and Ce^4+^ states, respectively, obtained from Gaussian fitting

Figure 2a shows the Ce 3*d*_5/2_ XAS spectrum of the dry SDC thin film. The Ce 3*d*_5/2_ spectrum corresponds to the transition from the Ce 3*d*_5/2_ core level to the unoccupied Ce 4*f* states. The overall shape and peak position of the thin film were in good agreement with those of the CeO_2_ thin film [3, 4, 33]. Using Gaussian fitting, the peak positions of on-1 and on-2 indicated in the spectrum were estimated to be Ce^3+^ and the peak positions of on-3 was estimated to be Ce^4+^. This result indicates that the SDC thin film has mixed valence states of Ce^4+^ and Ce^3+^. There was no significant difference in the spectrum shapes between the dry- and wet-annealed thin films, indicating that the resolution of the XAS method is not sufficient to detect the effect of proton insertion on the electronic structure. Therefore, as shown in the next section, we applied the resonant PES method to the SDC thin films, which method has significantly better resolution.

Figure 2b shows the resonant PES spectra of the as-deposited and wet-annealed SDC thin films, measured at photon energies indicated by on-1, on-2, and on-3 in Fig. 2a. The PES spectra examined in this study reflect the surface electronic structure, since the mean free path of a photoelectron is less than 2 nm [34]. The intensities of these spectra were normalized by the acquisition times and beam current. The spectral intensities are resonantly enhanced at on-1, on-2, and on-3. The PES spectra are explained as follows: (i) the resonant PES spectra measured at on-1 and on-2 have peaks at a binding energy of ~ 2.0 eV, which corresponds to the Ce^3+^ state (3*d*^9^4*f*^1^*L*) hybridized with the O 2*p* state. Here, *L* is ligand hole in the O 2*p* state; (ii) the spectra measured at on-3 has a peak at a binding energy of ~ 4.3 eV, which corresponds to the Ce^4+^ state (3*d*^9^4*f*^0^) hybridized with the O 2*p* state. In the as-deposited thin film, the abundance ratio of Ce^4+^ at ~ 4.3 eV and Ce^3+^ at ~ 2.0 eV is estimated to be 88:12. This result provides additional evidence for the mixed-valence states of Ce^4+^ and Ce^3+^, as shown in Fig. 2a. The peak intensity of Ce^3+^ at ~ 2.0 eV is lower in the wet-annealed thin film, indicating that the oxygen vacancies are occupied by oxygen ions in a wet atmosphere.

**Fig. 3:**
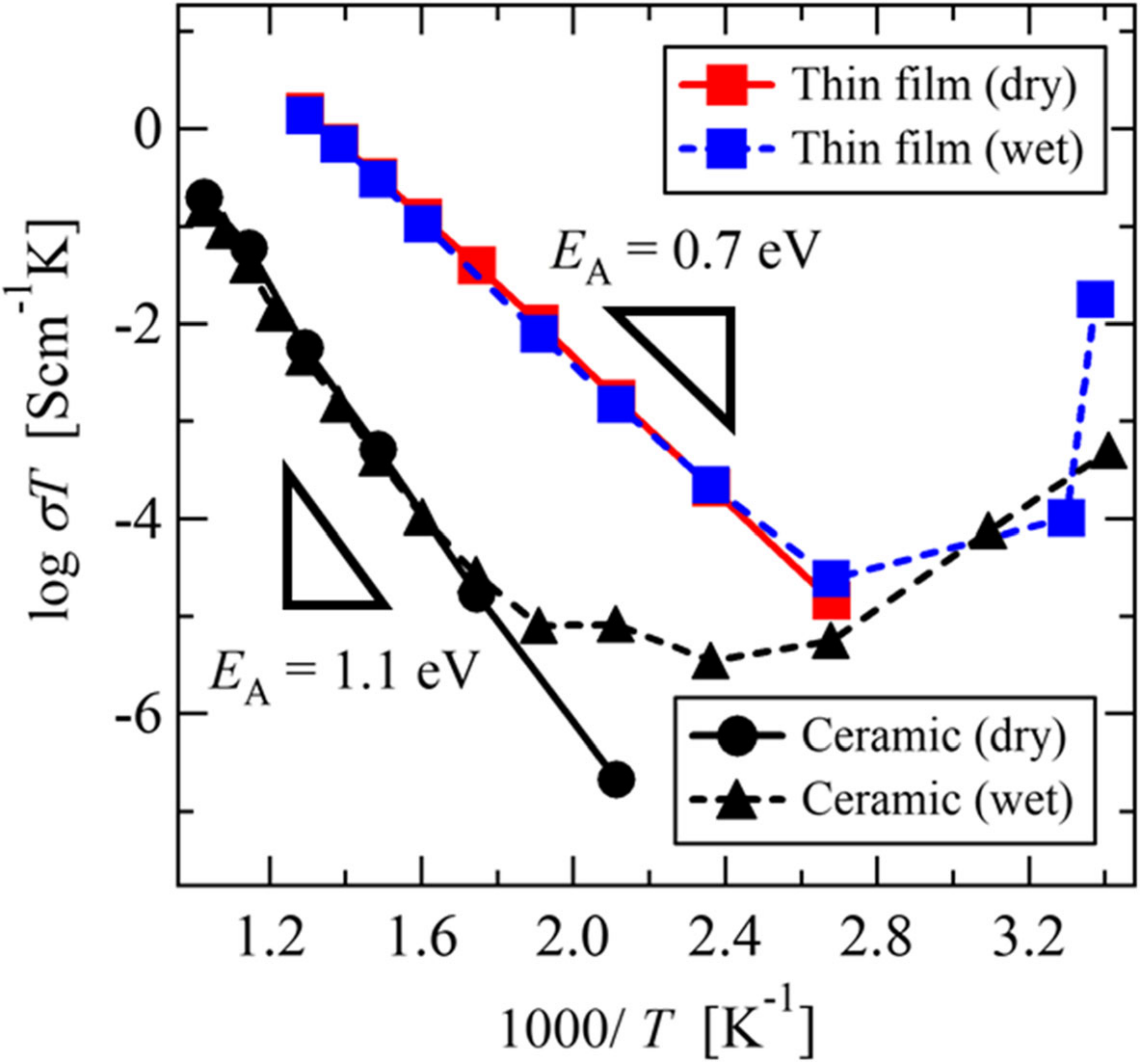
Arrhenius plots of the electrical conductivities in the in-plane of the SDC thin films and bulk ceramics, measured in dry and wet atmospheres

Figure 3 shows the Arrhenius plots of the electrical conductivities of the SDC thin films and bulk ceramics measured in dry and wet atmospheres. In the dry atmosphere, the SDC thin film and bulk ceramic exhibit Arrhenius-type thermal activation behaviors over the whole temperature range. The activation energies (*E*_A_) of the thin film and bulk ceramic are 0.70 and 1.1 eV, respectively. The conductivity of the polycrystalline SDC ceramic was two orders of magnitude lower than that of the SDC thin film, due to the influence of grain boundaries. The same activation energy and similar conductivity have been reported for Gd-doped CeO_2_ polycrystals and thin films [4, 18].

In contrast, due to the proton migration, the conductivities of the thin film and the bulk ceramic measured in a wet atmosphere gradually increase as the temperature decreases to below 100 and 250 °C, respectively. In particular, the increase in the conductivity ratio was more marked in the thin film. Single crystals and micropolycrystalline CeO_2_ do not exhibit proton conductivity, but since such proton conduction is caused by absorbed protons at the surface, nanopolycrystals and porous CeO_2_ do exhibit proton conductivity [19, 20].

In general, the room temperature surface proton conduction of fluorine-type oxides such as CeO_2_ or YSZ is explained by the Grotthuss mechanism [14–18]. According to this mechanism, physisorbed H_2_O forms OH^−^ and H_3_O^+^ ions on the surface at room temperature and the H_3_O^+^ proton transfers from one H_2_O molecule to a neighboring H_2_O molecule, as in the following reaction:

2 $$ {\mathrm{H}}_2{\mathrm{O}}^{+}+{\mathrm{H}}_2\mathrm{O}\to {\mathrm{H}}_2\mathrm{O}+{\mathrm{H}}_2{\mathrm{O}}^{+} $$

Similar behavior was observed in the CeO_2_ and YSZ thin films and bulk ceramic [14–24].

**Fig. 4:**
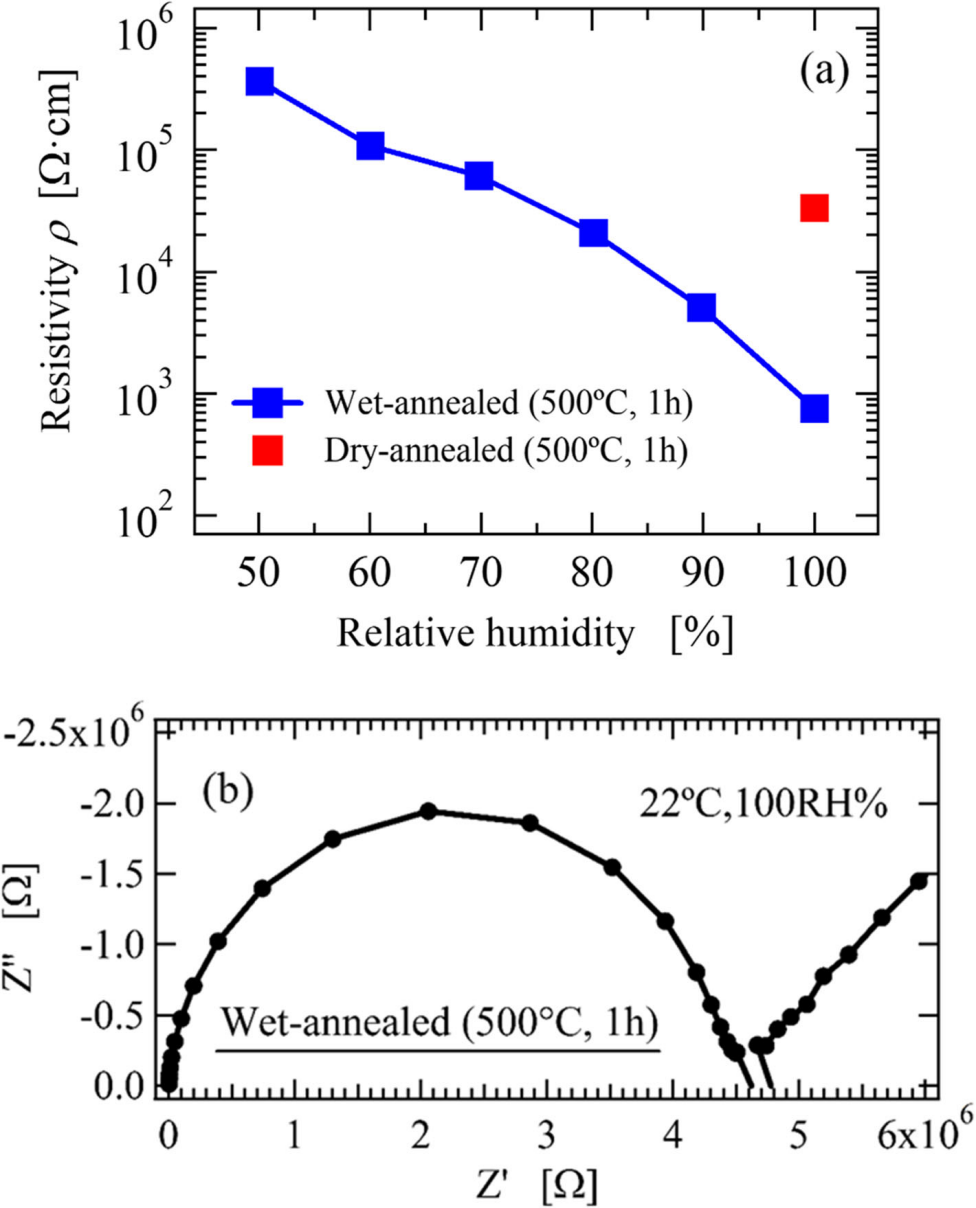
**a** The relative humidity dependence of the wet-annealed SDC thin film and **b** Cole-Cole plots of the wet-annealed SDC thin film, measured in 100 RH% wet air at 22 °C

**Fig. 5:**
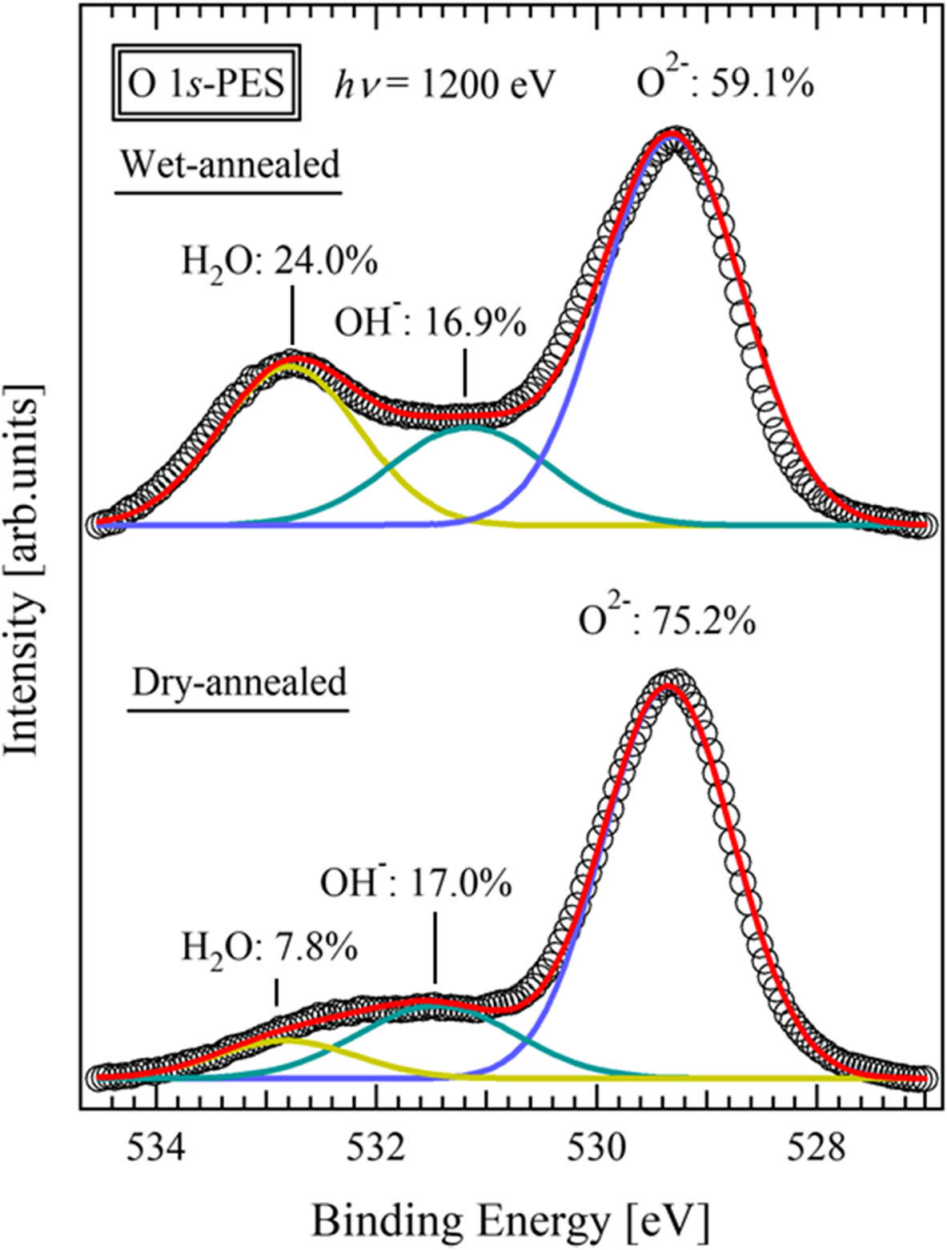
PES spectra of the O 1*s* core level of dry- and wet-annealed thin films. The blue, green, and yellow curves are the O^2−^ in the lattice site, and OH^−^ and H_2_O on the surface, respectively, obtained from Gaussian fitting

The dependence of relative humidity on the resistivity of the wet-annealed SDC thin film, at room temperature, is shown in Fig. 4a. The resistivity decreased greatly as the relative humidity increased and decreased by three orders of magnitude when the humidity was increased from 50RH% to 100RH%. The dramatic increase in the conductivity of the SDC thin film at room temperature, as shown in Fig. 3, is due to the increase in physisorbed water on the SDC surface as the relative humidity increases. The red plot shows the resistivity of dry-annealed SDC thin film measured in a 100RH% wet atmosphere at 22 °C, which resistivity was two orders of magnitude higher than that of the wet-annealed SDC thin film. This indicates that the proton absorption at the SDC surface, by wet-annealing, increases the surface proton conductivity. Figure 4(b) show the Cole-Cole plot of the wet-annealed thin film measured at 22°C. The spectrum is shown in order to distinguish the bulk resistance and electrode interface resistance at the low temperature region shown in Fig. 3. The wet-annealed thin film exhibits one semicircle and the tail of a second semicircle, indicating that the conducting carrier is surface conducting protons. Figure 5 shows the O 1*s* PES spectra of the dry- and the wet-annealed thin films. Both exhibited a double-peak structure and a sharp peak at ~529.5 eV, which corresponds to O^2-^ in oxygen sites. On the other hand, the positions of the weaker peaks are different, and can be interpreted as follows: (i) the broad peak at ~532 eV in the as-deposited thin film corresponds to the OH- absorbed at the surface created by chemisorbed water.; and (ii) the peak at 533 eV in the wet-annealed thin film corresponds to H_2_O molecules physisorbed at the surface [35]. The same peak structures have been reported in YSZ thin film with surface proton conduction at room temperature [15, 36]. The peak ratio of physisorbed H_2_O was increased from 7.8% to 24% by wet-annealing. Thus, the increase in conductivity by wet-annealing, shown in Fig. 4, reflects an increase in the physically adsorbed water molecules at the SDC surface. A proton conductivity of 5.98×10^-5^ S⁄cm was achieved at room temperature in the preferentially oriented thin film, which is two orders of magnitude higher than that of polycrystalline ceramics. Such conductivity is applicable to practical devices [26–30]. Most notable was the ~10^-2^ S/cm proton conductivity exhibited in a high humidity atmosphere, as shown in Fig. 4(a), which is considerably higher than the highest proton conductivities reported so far; approximately ~10^-4^ S/cm for Gd-doped CeO_2_ thin films [19] and ~10^-6^ S/cm for Gd-doped CeO_2_ polycrystals [18]. Such high proton conductivity is considered to derive from two features of the preferentially oriented SDC thin film with oxygen vacancy. The first feature is high water adsorbability on the SDC (111) surface. In the O1*s* PES spectrum, 16.9% of the detected oxygen was attributed to chemically adsorbed water and 24% was attributed to physically adsorbed water. This means that there are layers of physisorbed water on the SDC surface that can acts as proton conducting paths. The second feature is the dissociation of adsorbed water at the SDC (111) surface. The reduced CeO_2-δ_(111) surface promotes the dissociation of water molecules and the formation OH^-^ and H^+^, which contribute to proton conduction [37, 38]. Dissociated protons can migrate through a physically adsorbed water layer by the Grotthus-mechanism. Therefore, the preferentially oriented SDC thin film contributed to such high proton conduction.

## Conclusion

We have studied the surface proton conduction of SDC thin films prepared by RF magnetron sputtering. The prepared SDC thin film was preferentially oriented in the [111] direction, and the surface of the film was reduced by Sm doping. From the Ce 3*d*, Sm3*d*, and O1*s* core level PES spectra, the composition of the SDC thin film was calculated to be Ce_0.858_Sm_0.142_O_1.912_.

The conductivity of the thin film is higher than that of bulk ceramic due to its preferential orientation, which is not affected strongly by grain boundaries. Due to water condensation on the SDC surface, the proton conductivity of the wet-annealed SDC thin film increases as the temperature is decreased to below 100 °C, although it exhibits oxygen ion conduction above 100 °C.

A high proton conductivity of ~ 10^−2^ S/cm was achieved in a high-humidity atmosphere, at room temperature. This is due to the characteristics of the preferentially oriented SDC thin film with oxygen vacancies. The presence of physisorbed water layers on the SDC surface, indicated by the O1*s* PES spectrum, acted as a proton-conducting path by the Grotthuss mechanism. The SDC (111) surface with oxygen vacancy promoted water dissociation and the formation of protons. Generated protons on the SDC (111) surface were conducted through the physisorbed water layer, and a high proton conductivity was achieved.

## Data Availability

The data generated during and/or analyzed during the current study are available from the corresponding author by request.

## Abbreviations

*d*_111_: Spacing of the (111) plane
*E*_A_: Activation energy
EDL: Electric double layer
EDLT: Electric double-layer transistor
*E*_g_: Energy gap
PES: Photoemission spectroscopy
RF: Radio frequency
RH: Relative humidity
SDC: Sm-doped CeO_2-*δ*_
SOFC: Solid oxide fuel cell
XAS: X-ray absorption spectroscopy
XRD: X-ray diffraction
YSZ: Y-stabilized ZrO_2_
